# The Potential for Reducing the Number of Pneumococcal Conjugate Vaccine Doses While Sustaining Herd Immunity in High-Income Countries

**DOI:** 10.1371/journal.pmed.1001839

**Published:** 2015-06-09

**Authors:** Stefan Flasche, Albert Jan Van Hoek, David Goldblatt, W. John Edmunds, Katherine L. O’Brien, J. Anthony G. Scott, Elizabeth Miller

**Affiliations:** 1 Department of Infectious Disease Epidemiology, London School of Hygiene and Tropical Medicine, London, United Kingdom; 2 Immunisation, Hepatitis and Blood Safety Department, Public Health England, London, United Kingdom; 3 Immunobiology Unit, University College London, London, United Kingdom; 4 International Vaccine Access Center, Johns Hopkins Bloomberg School of Public Health, Baltimore, Maryland, United States of America

## Abstract

Stefan Flasche and colleagues consider whether the number of PCV doses in the infant schedule could be reduced without compromising its public health benefit in high-income settings.

**Summary Points**

- In high-income countries, pneumococcal conjugate vaccines induce strong herd protection that leads to near elimination of vaccine-type disease in vaccinated and unvaccinated alike.
- In settings with minimal exposure to pneumococcal vaccine types, individual protection from pneumococcal conjugate vaccine (PCV) is rarely required, and the majority of disease episodes are prevented by controlling vaccine-type transmission.
- Following the control of pneumococcal vaccine-type disease and colonisation through vaccination, a PCV schedule with a single priming and a booster dose may be sufficient to sustain that control at reduced costs and should be evaluated.

## Introduction

The most efficient use of limited health resources is an important principle of public health policy. For vaccine policy, this means balancing health, economic, and sociologic considerations to employ the best vaccine products and optimal schedules. However, the optimal schedule for vaccine introduction might not be optimal once indirect vaccine effects are established in the community. Persisting with dosing schedules that aim at the direct protection of individuals in settings where the additional benefit of direct protection is limited through the presence of herd effects may prevent inclusion of additional vaccines into the vaccination programme and consume resources that could be spent more efficiently elsewhere. Here we discuss how the substantial decline in transmission of strains targeted by the vaccine (vaccine-type pneumococci) in high-income countries following pneumococcal conjugate vaccine (PCV) introduction presents an opportunity to investigate if the number of PCV doses in the infant schedule could be reduced without compromising its public health benefit.

## Current PCV Schedules

A seven-valent PCV (PCV7), providing protection against the most common serotypes causing pneumococcal disease in high-income countries, was licensed in the United States and recommended for use in the infant immunisation schedule in 2000. The licensing schedule consisted of three priming doses at 2, 4, and 6 months of age and a booster dose at 12 to 15 months (3+1 schedule). Subsequent PCV trials demonstrated efficacy when administered without the booster dose (3+0 schedule) and aligned with the World Health Organization’s (WHO) Expanded Programme on Immunization (EPI) schedule [1]. Hence, the 3+0 schedule is adopted mostly by low- and middle-income countries that tailor their vaccination programmes to the EPI schedule.

In the United Kingdom, PCV7 was introduced with a two-dose primary series, at 2 and 4 months of age, and a booster dose at 13 months (2+1 schedule). This was adopted on the grounds of programmatic simplicity, reduced costs, and generally similar immunogenicity to the 3+1 schedule [2]. Motivated by similar considerations, including the need to create space for other vaccines in a crowded primary series, other high-income countries have adopted a 2+1 PCV schedule. Disease surveillance has demonstrated that 2+1 and 3+1 schedules both induce strong indirect protection reducing the incidence of vaccine-type colonization and consequent invasive pneumococcal disease (IPD) almost to the point of elimination within the community. Hence, the marginal superiority of the 3+1 schedule for direct protection of infants is scarcely measurable at the population level [3].

## Considerations for a Reduced PCV Schedule

As the strong herd effects that are induced by both a 3+1 and a 2+1 schedule resulted in a similar impact on IPD, the potential for further reduction of the primary series to a single dose (1+1) should now be assessed for settings with a mature vaccination programme, where vaccine-type carriage is largely eliminated in the community, high vaccine coverage of both doses can be assured, and careful monitoring is feasible. Other two-dose schedules may be similarly effective; however, we focus our argument on the simplest programmatic option, i.e., the removal of an existing dose. A schedule with fewer doses in the primary schedule could substantially reduce the costs of pneumococcal immunisation and make space for other vaccines in crowded infant vaccination programmes in high-income countries. A single dose in infancy induces lower antibody responses and provides less protection against disease and carriage than a two- or three-dose primary series [4]. In mature vaccine programmes, however, individual protection is rarely required because the probability of exposure to vaccine-type infection is low. For example, 8 years after the introduction of PCV7 in the US, the relative prevalence of vaccine-type pneumococcal carriage among children in Massachusetts fell from 36% to 2%, and vaccine-type IPD was virtually eliminated [3,5].

The success of a 1+1 PCV schedule is contingent on its ability to sustain established herd protection, which, in turn, depends on the relative contribution made by different doses in the schedule to reducing vaccine-type transmission. The magnitude of reduced transmission for each serotype, and consequently the magnitude of serotype-specific herd protection against pneumococcal disease, is a result of several factors, including the vaccine efficacy against carriage acquisition, the duration of vaccine protection, the prevalence of carriage, and the number of contacts of individuals. In high-income settings, children aged 1–4 years have the highest carriage prevalence and have high contact frequencies implicating them as strong drivers of pneumococcal transmission. Protecting this age group by vaccination, therefore, has a disproportionately powerful influence on herd protection. Given the relatively lower contact frequencies of infants, protection against carriage in the first year of life is less likely to contribute to overall herd protection. Hence, the key epidemiologic question is, would a reduced primary compromise the present levels of herd protection by reducing the vaccine efficacy of the booster dose against acquisition of carriage?

The relative impact of a booster dose on carriage following a reduced primary series of one versus two doses has not been studied. Among Israeli toddlers, acquisition rates for pneumococcal carriage and serotype-specific anticapsular immunoglobulin G (IgG) were compared both before and after receipt of a booster dose at 12 months of age between groups of children who were administered two or three primary doses [6]. After priming with two instead of three doses, vaccine-type acquisition rates were lower in the 18 months post booster, and antibody concentrations at 1 and 7 months after boosting were similar. In the UK, antibody levels after the PCV7 booster were significantly higher for serotypes 4, 9V, and 14 in children primed with two instead of three doses, but lower for 6B and 23F [7]. By extension, further reducing the primary series to a single PCV dose would not necessarily impair the postbooster immune response, but this remains to be established. Notably, the total quantity of antigen used for priming with *Neisseria meningitis* type C conjugate vaccine is negatively correlated with the magnitude of the booster response [8].

Assuming that a 1+1 PCV schedule is found to maintain herd protection through the control of vaccine-type carriage, the optimum timing of the programmatic switch to a reduced primary series would need careful consideration. Although there would be little risk of additional IPD cases associated with a reduced primary series once vaccine-type pneumococcal disease has been eliminated in the population, the schedule switch may be cost-effective before that. An example of this kind of reasoning is given by the decision in the UK not to expand the indication for PCVs to adults at increased risk for pneumococcal disease although there was residual circulation of vaccine types in the population in 2012 [9]. Other recent policy decisions illustrate the acceptance and importance of incorporating herd protection in the evaluation of vaccine programmes. Maintaining herd protection without increasing programmatic costs was the defining factor in the decision of the UK to move the second dose of the three-dose monovalent meningococcal C conjugate vaccine schedule from infancy to adolescence, when carriage prevalence is likely to be highest [10]. A shift in vaccination programmes towards increasing reliance on indirect protection requires informing and educating both vaccinators and vaccinees on the associated benefits and risks. This needs to include discussions on whether any increase in individual risk and associated equity issues is acceptable if this frees resources that can otherwise be used more effectively to benefit public health. These have been central points in the recent discussion among the Advisory Committee on Immunization Practices in the US on a potential reduction of a four-dose PCV schedule to a three-dose schedule [11].

## The Way Forward

Before the implications of a policy shift to a reduced dose PCV schedule can be weighed objectively, key evidence gaps need to be filled. These include (1) the relative impact of a one- or two-dose primary series on the vaccine effectiveness of the booster dose against carriage—measured in terms of magnitude and duration, (2) the relative impact of one or two primary doses on protection against disease before the booster, and (3) the impact of age of administration of a single primary dose on (1) and (2). Given the virtual elimination of vaccine-type carriage in high-income countries that have introduced PCVs, the predominant question (1) is intractable in these settings.

Correlates of protection (CoPs) offer a potential alternative to the assessment of carriage as a study end point. Higher valent PCVs have been licensed based on noninferiority immunogenicity studies using an aggregated threshold of 0.35 μg/mL IgG antibody as a CoP against IPD, although this threshold likely is an oversimplification [4]. Serum immunogenicity as a CoP against pneumococcal carriage is less well established. As mucosal immunity is central to protecting against carriage acquisition and reducing density of colonization, the measurement of systemic antibodies is recognised as a proxy for what is likely occurring at the mucosal surface. The impact of PCV at the mucosal level is dependent on both the number and timing of PCV doses [12]. A putative protective threshold of 4–5 μg/mL IgG antibody has been suggested for antibodies acquired by PCV immunisation or natural infection [13], similar to the threshold found for *Haemophilus influenzae* type B (Hib) conjugate vaccine protection against Hib colonisation. However, the reverse cumulative distribution of pooled antibody concentration [4] in combination with an observed vaccine efficacy against colonisation of 40%–50% suggests that 2 μg/mL IgG antibody may suffice as a protective threshold for colonisation. If a 1+1 schedule is found to induce inferior postbooster protective serum antibody concentrations against colonisation transmission, dynamic models can help to assess the effect of reduced herd protection on the risk for pneumococcal disease in the community. Also, rigorous postimplementation surveillance of pneumococcal colonisation and disease, in particular among risk groups, will be essential to immediately detect a potential resurgence of vaccine-type circulation in the community.

Currently, the conditions for regimen simplification, and therefore the potential benefits, are limited to high-income countries. The additional epidemiologic and programmatic considerations around a two-dose PCV schedule in low-income countries are complex and beyond the scope of this commentary. However, successful implementation in high-income countries would support the evidence base for a reduced PCV series and may incentivise the evaluation of alternative PCV schedules in low-income countries that could eventually lead to a much welcomed reduction in the costs of the PCV programme.

In conclusion, evidence from high-income countries, which have accumulated up to 15 years of routine PCV use, consistently demonstrates that most vaccine-type pneumococcal disease has almost been eliminated among both unvaccinated and vaccinated children [3]. The changing epidemiology of the pneumococcus now invites us to consider whether the successful elimination of vaccine-type disease could be retained in a childhood immunisation schedule with fewer PCV doses. There is a compelling argument to now generate the evidence needed to evaluate whether such a change in the PCV schedule is beneficial.

## Abbreviations

CoP: correlates of protection
EPI: Expanded Programme on Immunization
Hib: *Haemophilus influenzae* type B
IgG: immunoglobulin G
IPD: invasive pneumococcal disease
PCV: pneumococcal conjugate vaccine
PCV7: seven-valent PCV
WHO: World Health Organization
